# Intervention strategies with 2D cellular automata for testing SARS-CoV-2 and reopening the economy

**DOI:** 10.1038/s41598-022-17665-3

**Published:** 2022-08-05

**Authors:** Igor Lugo, Martha G. Alatriste-Contreras

**Affiliations:** 1 Centro Regional de Investigaciones Multidisciplinarias (CRIM), National Autonomous University of Mexico, 62210 Cuernavaca, Mor. Mexico; 2 Centro de Ciencias de la Complejidad (C3), National Autonomous University of Mexico, 04510 Mexico City, Mexico; 3 Facultad de Economía, National Autonomous University of Mexico, 04510 Mexico City, Mexico

**Keywords:** Infectious diseases, Computational science, Socioeconomic scenarios

## Abstract

During the period of time between a new disease outbreaks and its vaccine is deployed, the health and the economic systems have to find a testing strategy for reopening activities. In particular, asymptomatic individuals, who transmit locally the COVID-19 indoors, have to be identified and isolated. We proposed a 2D cellular automaton based on the SI epidemic model for selecting the most desirable testing frequency and identifying the best fitting size of random trails on local urban environments to diagnose SARS-CoV-2 and isolate infected people. We used the complex systems approach to face the challenge of a large-scale test strategy based on urban interventions, starting with first responders and essential workers. We used the case of Mexico to exemplify a credible and intelligent intervention that reduces the virus transmission and detects economic and health costs. Findings suggest that controlling and stopping the virus transmission in a short period of time are possible if the frequency of testing is daily and the percentage of random samples to be tested is at least 90%. This combination of model parameters represents the least expensive intervention compared to others. Therefore, the key for a national testing-isolating strategy is local interventions.

## Introduction

In the current pandemic of the coronavirus disease 2019 (COVID-19), the health and the economic systems of all nations have been stressed to their limits and started to collapse. This situation has showed the vulnerability and fragility of such systems to expected external shocks^1–3^. The economic activity in each country has decreased due to the coronavirus restrictions, in particular the national lockdowns. It slowed the severe acute respiratory syndrome coronavirus 2 (SARS-CoV-2) transmission, but affected the consumer behavior thus decreasing demand, which, as consequence, slowed down or stopped the flow of resources in the supply chain. The main expected effect has been the increasing unemployment. For example, in the USA the unemployment has showed an amount of 21.0 millions American jobs lost, a rate of 13.3% in May^4^. In Mexico, what is even worse is the lack of accurate data about the impact of such a lockdown in the economy. According to the Occupation and Employment Survey (ETOE in Spanish) of the National Institute of Statistics and Geography (INEGI in Spanish), from March to April 2020, the unemployment increased 12.5 millions^5^. Therefore, the economic conditions point out the importance of opening the economy as soon as possible, but it presents a higher health cost to the population. In particular, opening the economy without a vaccine presents a social dilemma between the economy vs the health of the nation. This idea was clearly stated by Anthony Fauci, NIH National Institute of Allergy and Infectious Diseases Director, who said “How many deaths and how much suffering are you willing to accept to get back to what you want to be some form of normality sooner rather than later?”^6^ Days before this statement, important ideas came from the Nobel Laureate economist Paul Romer^7^, who stated “mass testing is the key to reopening the economy”, and, later on, Osterholm^8^ and his colleague of the Center for Infectious Disease Research and Policy (CIDRAP)^9^ proposed a smart testing framework. Then, such a dilemma is solved based on a sequential testing and isolating strategies that can identify and lock down those who are infected, in particular asymptomatic individuals. Therefore, we proposed a theoretical approximation based on cellular automata (CA) and the Susceptible-Infectious (SI) epidemic models to explore local testing scenarios—small workplaces and communities—for returning to work and keeping the contagion slow. Local strategies are the most appropriate interventions for identifying asymptomatic individuals and keeping track of the health and the economic costs.

Since the COVID-19 outbreak, the number of studies about modeling epidemics has been considerable. In particular, the epidemic compartmental models have been used to understand the dynamics of the contagion. These type of formulations are related to ordinary differential equation (ODE) models that describe the dynamic of the system in question^10,11^. Such models are commonly used to cover the SI (susceptible and infectious), SIR (susceptible, infectious and recovered), and SEIR formulations (susceptible, exposed, infectious and recovered)^12,13^. One of the problems with this type of models is to model spatial transmissions at the local community level^14^. On the other hand, there are statistical approximations for understanding the behavior of the epidemics^15^. Based on fitting curves to empirical data, scientists have suggested possible models that replicate such behaviors. However, the problem with this approximation is the need for data, i.e., the more data we have, the more accurate findings, interpretations, and recommendations we get. Therefore, these approximations have been appropriated for studying the dynamic of contagion without being in a period of serious danger. However, far too little attention has been paid to understand the mechanism for controlling or stoping the virus transmission just in the moment when the outbreak happened.

In accordance with such research studies about modeling epidemics, we used the complex systems approximation and the economics field to explore testing scenarios. These scenarios consider a test in which the infection is diagnosed in a period of 15 min at point-of-care locations, for example the current SalivaDirect and the reverse transcriptional loop-mediated isothermal amplification (RT-LAMP)^16–19^. However, there are other test options—the antibody test, computed tomography (CT) scans, and reverse-transcriptase polymerase chain reaction (RT-PCR) viral test—that diagnose SARS-CoV-2^20,21^. Based on such viral tests or similar tests in the market and the well-known 2D CA models^22–24^, we applied the discrete, spatial formulation of the SI model because it describes the simplest form of contagion without immunity, treatment, and vaccine^25–27^. Then, we identified the time interval to apply the COVID-19 test and to select a subset of randomly chosen individuals for testing into local environments—i.e., hospitals, companies, schools, academic departments, or similar others. In line whit this idea, Larremore et al.^28^ has suggested a regular testing frequency, even with low-sensitivity tests, for identifying the asymptomatic COVID-19 patients. Even though our approximation is easily generalized to other countries due to its vital importance, we used the case of Mexico because we are aware of the unsafe health conditions when reopening the economy without a vaccine and the flawed interventions to face the national health crisis. For example, the federal government has falsely claimed that the risk of transmission of an asymptomatic person is less than the symptomatic individual^29–31^, meanwhile the scientific evidence has showed the contrary^32–35^. To date (July 1, 2020), Mexico has showed an increasing trend in confirmed cases and deaths of COVID-19, and the federal interventions do not consider a testing strategy to control and stop the virus transmission of asymptomatic infected individuals as one of its main goals^36–40^. However, in May 31, 2020, the federal government restarted the economic activity in the country. Therefore, it is evident that without a vaccine in the short term, people of Mexico, particularly in urban areas, will be exposed to a higher risk to get SARS-CoV-2 and, hence, worsen the economic activity.

This study focuses on showing a simple theoretical^41^, local model to understand and to plan the best strategy to restart activities taking care of the health of people and considering the economic costs. In particular, during the time period between the spread of the disease and the production and distribution of its vaccine, we proposed an action plan to support and guide the health and the economic systems. Our main goals are to control and stop the virus transmission before all people get infected and to identify the economic cost of the intervention. We are interested in answering the following three questions: how to operationalize the return to work processes keeping the contagious rate lower? What is the best and the worst scenarios to open the economy? And what are their economic costs and benefits? To answer these questions, we followed Romer^7^ and Osterholm^8^ ideas in which different testing situations have to be analyzed to identify the best combination between the frequency of testing and the random sampling for selecting individuals. Based on this information, we used an exploratory data analysis for identifying trends and correlations to describe the best strategies to open the economy. In particular, we used a sensitivity analysis based on the sampling-based and scatterplot approaches to identify stable patterns in the dynamics of the 2D CA. To develop these analyses, we used the Python programming language to generate and analyze data, and we share it based on the open science principles^42^. We strongly believe that the testing capacity and its organization are the keys to reopen the economy and reduce the risk of contagion. We investigate the hypothesis that the scale of the community or workplace matters to reduce the time steps of transmission and to stop the spread of SARS-CoV-2. A small group of people concentrated indoors can be monitored and, consequently, the disease transmission can be better controlled in a few time steps, if the frequency of testing is daily and the size of random trails is at least 70% of the people. On the other hand, large groups of people partially concentrated indoors control worse the disease transmission in large time steps, if the frequency of testing is greater or equal than twice a week and the size of random trails is less than 70% of the people. The former is less expensive than the latter because the testing is intensive in time periods. In line with Prather et. al.^43^, Zhang et al.^44^, Dbouka and Drikakisb^45^, Swetaprovo et al.^46^, and Bazant and Bush^47^, we believe that, even though people follow health prevention measures to not get sick—i.e., social distancing and face cover—and the official guidelines to reopen the economy—i.e., flexible work hours and cleaning continuously surfaces, equipment, and other elements of work environment—asymptomatic individuals can infect a group of people concentrated indoors during a small time period of a day because of the airborne transmission. Therefore, small groups of first responders—workers related to the healthcare, transportation, cleaning, and food service systems—have to be tested and monitored constantly to guarantee the basic services to the population. Eventually, other essential workers—such as teachers, professors and researchers in academic places—have to be tested as well. Finally, testing everyone based on local communities.

This paper contributes to the scientific efforts for controlling the current pandemic of the SARS-CoV-2 and preparing us for the next pandemics to come. Identifying the frequency of testing and the random sample to be tested based on the size of local establishments, we can generate an effective, credible, and intelligent approach to save lives and return to work safely. We have to consider this type of approaches using the available tests until a vaccine is approved and deployed to everyone. Therefore, it is fundamental a testing strategy based on local environments, which are more accessible to coordinate efforts in the shortest time possible, to auto-organized the society fighting against the COVID-19, and to reopen the economy.

This paper is organized as follows: “Material” section describes the basic parameters to use in the 2D CA model; the “Method” section describes the CA based on the SI model and the testing-isolating rule; the “Result” section shows findings and their interpretations; finally, the “Discussion” section shows final remarks and suggestions.

## Materials

Since our study shows a theoretical approximation for identifying the intervention related to testing SARS-CoV-2 and reopening safely the economy, we use particular types of data for contextualizing and calibrating our 2D CA. In particular, we selected a particular rate of transmission, a viral test, and a randomized controlled trails (RCTs) approximation.

Firstly, we use the rate of transmission—the number of secondary infections from one infected individual—between 2 to 2.5 provided by the Mexican federal government and the Pan American Health Organization (OPS in Spanish)^48,49^. Next, we assume the use of a viral test that diagnoses SARS-CoV-2 around a period between 15 min or less in the same day of testing, and we suppose that the test shows 0% of false positives. In particular, we are interested in the SalivaDirect and RT-LAMP viral test. The cost of the former is between $27.26 and $90.87 mxn—from $1.29 to $4.30 usd—per test^50^, and the latter is between $$\$1200$$ and $$\$7500$$ mxn—from $$\$53$$ to $$\$332$$ usd^51^. We used the exchange rate provided by Morningstar, October 17, 2020—Corporate Actions data provided by Thomson Reuters. Finally, we use the RCTs approximation in our model to show its importance and practical implementation in local indoor environments for reducing the virus transmission^52^. For example, different sizes of workplaces, based on the number of workers indoors, can randomly choose individuals and keep track of their testing results and contact tracing to monitor data. Therefore, because of the scale of the analysis, our approach can identify the benefits and costs of the testing-isolating strategy. Benefits are in terms of the number of days for reducing or stopping the virus transmission and for implementing the strategy. Costs are in terms of monetary values and health disadvantages or infected people when implementing the strategy.

In addition, we used a second type of materials based on programming libraries. We applied the Python programming language using the following libraries: https://matplotlib.org/index.html, https://numpy.org/doc/stable/index.html, https://www.scipy.org/, random, and https://docs.python.org/3/library/pickle.html. We provide the programing code, the data, the 2D CA, and the results in our Open Science Framework (OSF) project (10.17605/OSF.IO/7E98T).

## Method

Despite the fact that there are different epidemic models^53–55^, the SI formulation describes the spread of infectious diseases without considering immunity, treatment, and vaccine. These characteristics are closely related to the current knowledge of the SARS-CoV-2 behavior^44^. The SI model is defined as followed:

1 $$\begin{aligned}&\frac{dS}{dt} = \frac{-\beta S I}{N} \end{aligned}$$

2 $$\begin{aligned}&\frac{dI}{dt} = \beta I (1-\frac{I}{N}) \end{aligned}$$

where S = susceptible, I = infected; $S + I = N$ is the constant population size of individuals in the model; and $\beta$ = contagious rate between *S* and *I*. Once *S* is changed to *I*, without a treatment, individuals stay infected. Its dynamic shows an exponential growth of *I*, meanwhile *S* shows an inverse shape of *I*. Therefore, this formulation shows a mathematical tractability advantage due to its continuous approximation^26^.

Based on this formulation, we used the discrete, spatial approximation based on a 2D CA because it can describe accurately the local indoors contagious process. This type of modeling has been applied not only to epidemics, but also to describe large-scale processes, such as the growth of cities^24^. Therefore, we used the epidemiology concepts of the SI model to describe the method and to interpret results, but we used the framework of the complex systems to build and contextualize the 2D CA.

In this section, we present the 2D CA baseline model in which its rules are formed by deterministic and stochastic transitional rules. The former rule specifies the contagious mechanism based on the rate of transmission. The latter rule describes the testing-isolating strategy to control the virus transmission. These rules identify how the scale—the total number of cells in the CA—affects the intervention strategy: the testing-isolating frequencies and the number of cells randomly selected to be tested. Next, we apply a sensitivity analysis sampling-based and scatterplot approaches changing the parameters related to scale, test frequency, and the number of people randomly chosen for testing. The resulted scatterplots are analyzed by the https://docs.scipy.org/doc/scipy/reference/generated/scipy.stats.spearmanr.html rank coefficient to test the sensitivity of the selected parameters. Finally, we identify the statistical distribution of total number of people tested per model specification to provide the economic cost of the intervention strategy. Those distributions are identified by using the Kolmogorov-Smirnov (KS) goodness-of-fit test^56^.

### Baseline CA

We proposed a model using a regular 2D tessellation as a grid, where the state of each cell *i* depends on three attributes: Susceptible (S), Infected (I), and Tested-Isolated (TI). Then, each cell has three states, $S_{i} = 0$, $I_{i} = 1$, and $TI_{i} = 2$. We selected the Moore neighborhood $\Omega _{i}$ defined by eight adjacent cells. The validity of using this type of neighborhood is that it resembles the radius of the personal space (PS) concept^57^. Following the idea of Hayduk^58^ a PS is formed by a circular area surrounding the person. Then, the PS is a fundamental concept to model spatial interactions at indoor activities. Furthermore, inside buildings and confined spaces the interior design and organization support and drive social interactions based on regular structures^59^. Depending on the assigned or unassigned workstation on a floor, the number of people around an individual is pretty much the same^60,61^. Therefore, the initial condition at time $t = 0$ are the following:

3 $$\begin{aligned} I_{i} = 1, \quad S_{i} = 0, \quad and \quad TI_{i} = 0 \end{aligned}$$

where $I_{i} = 1$ is located in the center of the array. In this model, every *t*, which is an iteration, describes a day. Then, the transitional rules at any time *t* are the following:

4 $$\begin{aligned} \begin{aligned}{}&\text {if} \quad S_{i}(t) = 0 \quad and \quad \sum _{k \subset \Omega _i} I_{i}(t) = 1 \quad \text {or } = 2 \quad \text {or } = 3 \\&\quad then \quad I_{i}(t +1) =1 \end{aligned} \end{aligned}$$

where Eq. (4) describes the rate of transmission based on the number of susceptible receptors in the neighborhood. We used from one to three active cells in the neighborhood as the unweighted infectious rate. Therefore, one infected cell can transmit the virus to its neighbors, from $S_{i} = 0$ to $I_{i} = 1$. Next, we defined the testing-isolating rule that depends on specific time frequencies (intervals) $t_{interval}$ and a random sample of cells to detect and isolate infected cells.

5 $$\begin{aligned} \text {If} \quad t&= t_{interval}: \nonumber \\&random\_sample = N \phi \nonumber \\&\text {For} \quad i \quad \text {in} \quad random\_sample: \nonumber \\&\quad \quad \text {If} \quad I_{i}(t) = 1 \quad \text {then} \quad TI_{i}(t+1) = 2 \end{aligned}$$

where $t_{interval}$ describes three types of values $\left\{ 1 = \text {daily }, 3 = \text {twice a week }, \text { and } 7 = \text {a week}\right\}$, and $random\_sample$ is the fraction of the population selected randomly—based on a uniform distribution—by $\phi$ to be tested. Then, if the cell is detected as infected *I* in $t +1$ it is isolated. This type of cell stays isolated throughout its life, i.e., in next iterations such cells were not considered in the computation. Then, in each $t_{interval}$, the number of cells associated with the random sample decreases because of the number of isolated cells. Therefore, the number of testing cells is collected to identify the total economic cost per model.

Next, we applied a sensitivity analysis based on scatterplots to show different scenarios when varying the size, testing-isolating intervals, and the percentage of people in the sample. In particular, we are searching for possible correlations between the testing intervals and the size of random samples that identify the best combination of parameters to reduce or stop the virus transmission in fewer time steps. We analyzed and selected the scenarios of interest that best describe the relationship between those parameters. Moreover, these resulted scatterplots are analyzed by the Spearman rank correlation, and we identify the statistical distribution associated with the total number of cells that were tested in each combination of model parameters. To identify the best fit of statistical distributions, we used the KS goodness-of-fit test.

## Results

To recapitulate and summarize what we have seen in previous sections, we present the main assumptions of our theoretical model. They describe accurately our main ideas and our expert intuition^62,63^ about an intervention based on local testing strategies. Then, the main assumptions are the following:

- Indoor environment. Spreading events in local situations related to the workplace of first responders. Even though persons follow the infection prevention and official guidelines for reopening the economy, the transmission of the virus is highly probable by spreading in aerosol and droplets.
- Short-term dynamics. Modeling the dynamics of the virus transmission in a short-term period (30 days maximum).
- No vaccine, immunity, and treatment. No vaccines including boosters available for getting seriously ill, being hospitalized, and dying.
- Local testing. Detecting in 24 h with a %0 of false positives the infection with SARS-CoV-2 based on any viral test.

**Figure 1:**
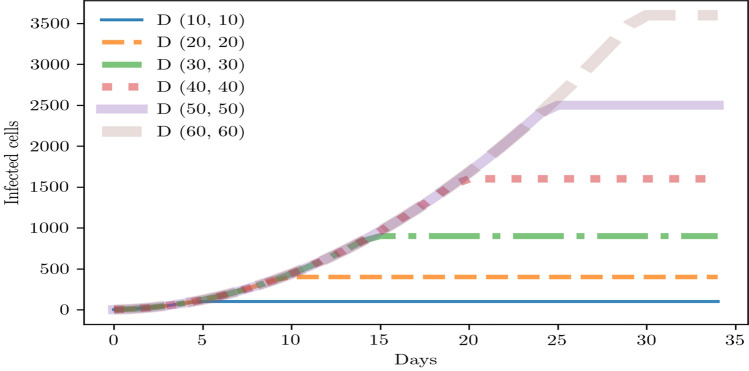
Virus transmission without any intervention in different array sizes. *D*( ) represents the dimension of the array. The starting point of lines with zero slopes describes the number of days in which all cells were infected.

In addition, we specify the simulation setting and parameters based on the rule (5). This set of combinations was selected because it showed stable patterns in its dynamics, and the other possible combinations showed a complete contagion of cells (see the OSF project for the full set of results). The latter are scenarios that we want to avoid due to their higher health cost. Then, the simulation setting and parameters are the following:

- No testing-isolating strategy: One asymptomatic person located at the center of the array spread the virus in several days. The number of cells depends on the array dimension. The array dimensions are the following tuples (10, 10), (20, 20), (30, 30), (40, 40), (50, 50), (60, 60). Testing frequencies and random sample testing are not defined. See the Fig. 1.
- Testing-isolating strategy: One asymptomatic person located at the center of the array spread the virus in several days. Three types of array dimensions are used (10, 10), (30, 30), (60, 60). Each of these cases uses the following testing frequencies and random sample testing: (Frequency: Daily, sample: 90% and 75%), (frequency: twice a week, sample: 75%). See Figs. 4, 5, and 6.

Initially, we present the effect of the virus transmission without any testing-isolating strategy in local indoor environments (Fig. 1). The spread of the virus depends on the number of cells in the array. After few time steps, all cells were infected. This relationship shows an increased curve with a positive trend. Therefore, small local indoor environments transmit faster the virus. On the other hand, large local indoor environments transmit the virus slower. At the end, people in both environments get totally infected.

**Figure 2:**
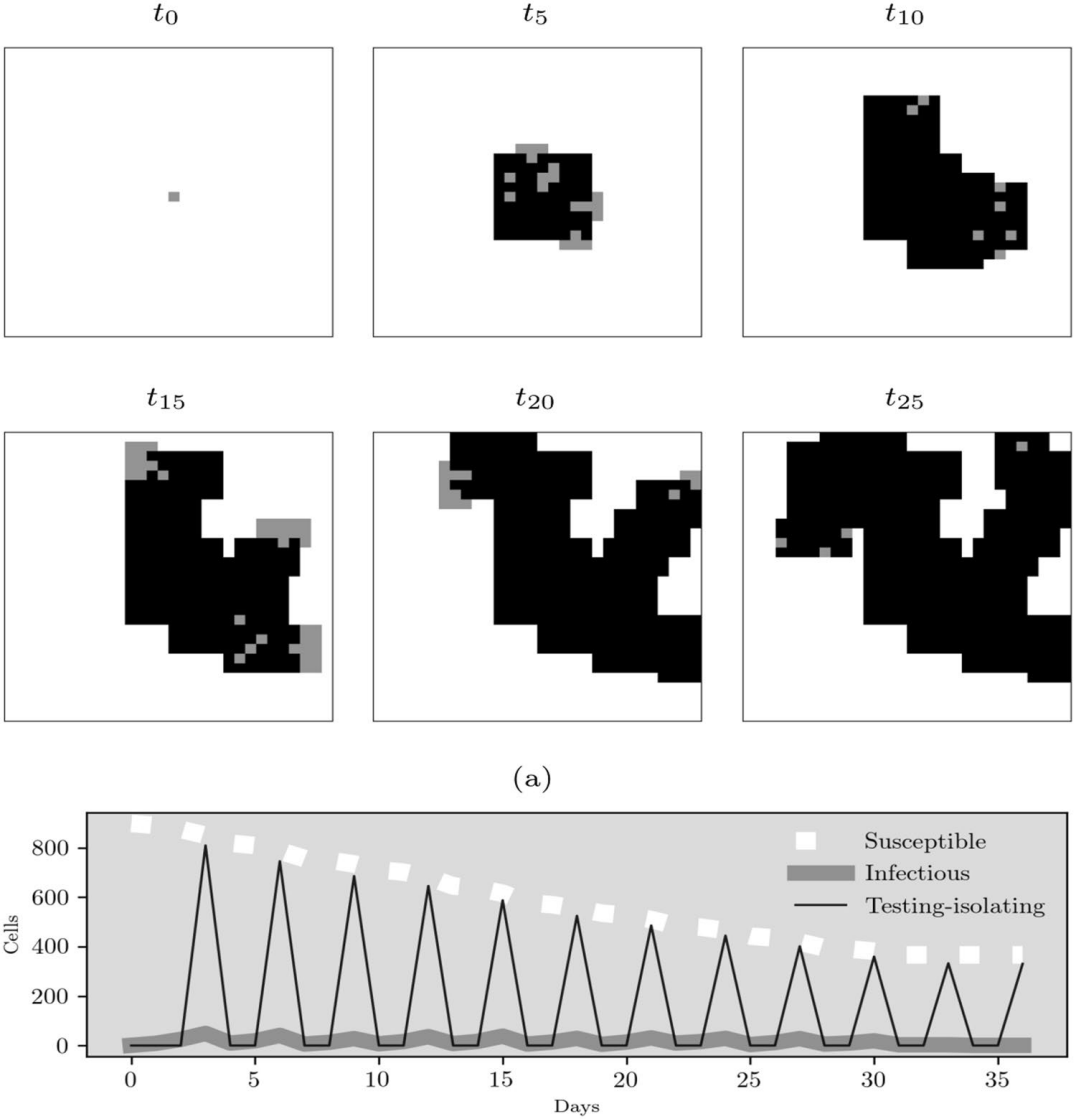
Dynamics of one CA simulation based on D(30, 30), a twice a week frequency test, and 90% of tested people. The simulation stops when the transmission was controlled, i.e., the number of infected cells is equal to zero.

In addition, we present one execution of our CA model with particular parameters to exemplify the performance of our code (Fig. 2). Figure 2 displays six frames that describe the dynamics of the CA. We observe in $t_{5}$ that the symmetry of the virus spreading was broken when the testing-isolating rule is introduced. As we mentioned in the rule (5), the random sampling instruction generates different transmission patterns in the model dynamics. Then, every time step when this rule is applied, we try to control or stop the virus transmission pattern identifying infected cells and isolate them. This goal is achieved when the infected cells were zero, in $t_{37}$. This is displayed in Fig. 2a that shows the dynamics of the susceptible, infected and tested-isolated cells. Therefore, this simulation shows the stop of the virus transmission before all cells get infected in larger time steps.

**Figure 3:**
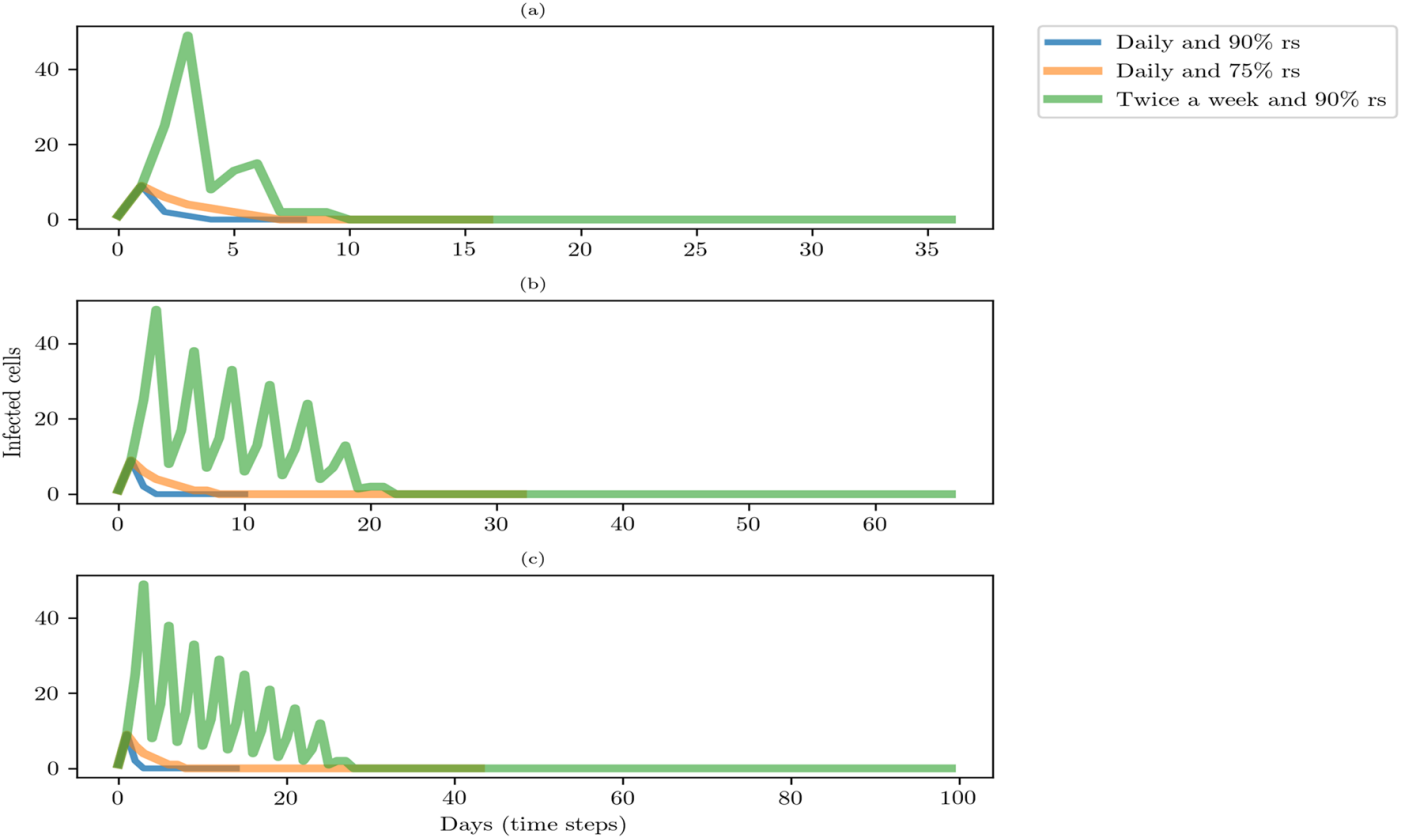
Trajectories of infected cells and number of days to control and stop the virus transmission. The computation of trajectories was based on the median of 10,000 model executions. (**a**) Array size of (10,10), (**b**) array size of (30, 30), and (**c**) array size of (60,60). Percentage of random sample (% rs).

Next, we show findings associated with the sensitivity analysis. Because of the computational expenses of executing the model 10,000 times in different array sizes, we selected three types: (10, 10), (30, 30), and (60, 60). They are a good representation of different types of indoor workplaces. Then, we selected a combination of parameter values that display the most significant and interesting behaviors. In particular, we showed the following combination of parameters: (S1) daily test and 90% of random sample, (S2) daily test and 75% of random sample, and (S3) twice a week and 90% of random sample. Based on this model specification, we present the dynamics of infected cells and the number of days-time steps-to control and stop the virus transmission (Fig. 3).

Figure 3 illustrates the rapid decrease in days of infected cells using the (s1) and (s2) testing-isolating strategies. On the other hand, the strategy (s3) shows a gradual decrease in days of infected cells. Therefore, the daily testing interval is preferable than the twice a week. The intensity of the test assists to identify faster infected cells. If the frequency of testing decreases, the identification of infected cells is slower and potentially harmful.

Next, we show findings associated with the sensitivity analysis based on scatter plots. We started with the array size of (10,10) and display the three testing strategies.

**Figure 4:**
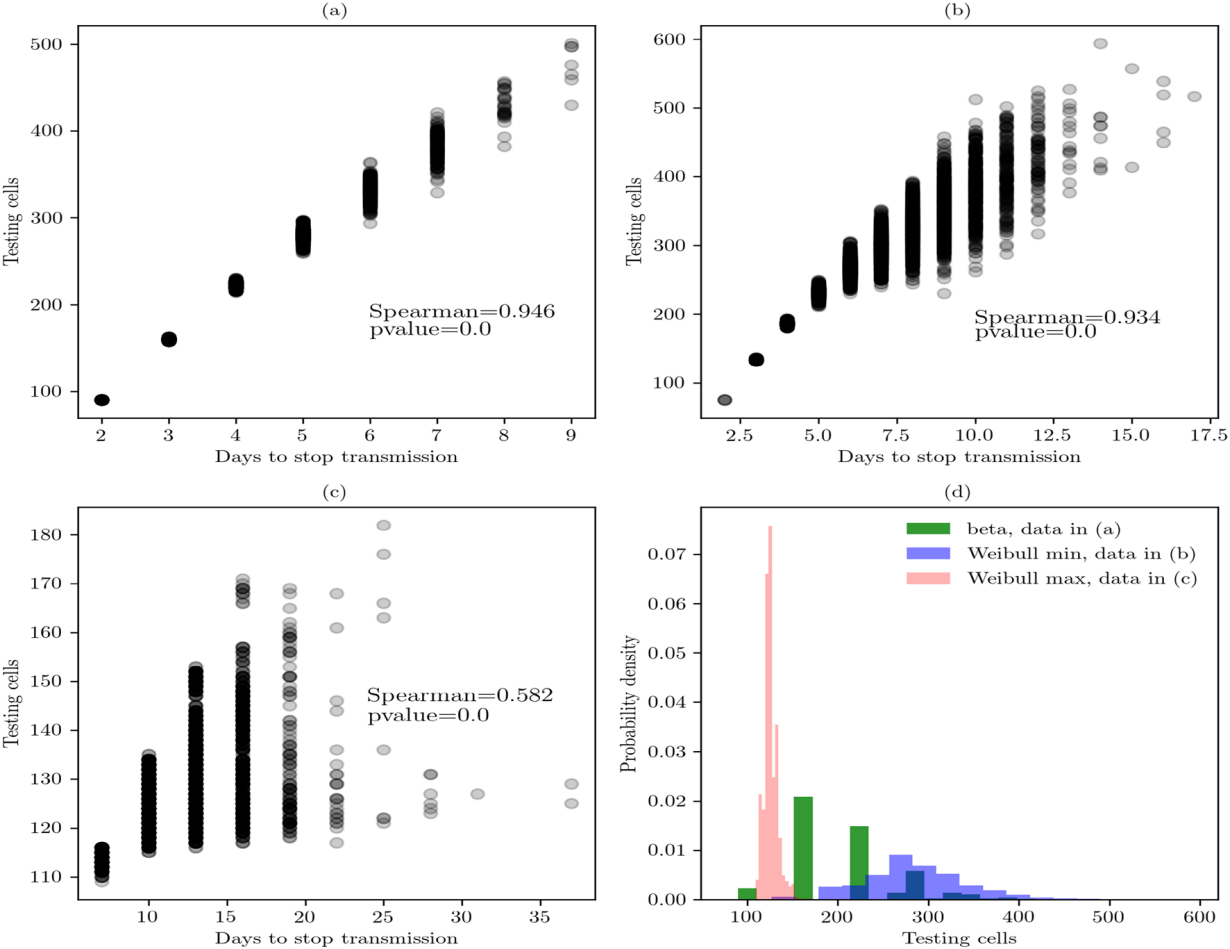
Sensitivity analysis based on scatterplots in the array of (10,10). (**a**) Daily testing and 90% of random sample. (**b**) Twice a week testing and 90% of random sample. (**c**) Daily testing and 75% of random sample. (**d**) Distribution fitting of the cost for the testing strategy in the three cases. Data in subfigure (**a**) is best described by a beta distribution that shows a probability density function of the form $f(x,a,b)=\frac{\gamma (a+b)x^{\alpha -1}(1-x)^{b-1}}{\gamma (a) \gamma (b)}$, for $0<x<1$, $a>0$, $b>0$. Data in subfigure (**b**) is best described by a Weibull min distribution that shows a probability density function of the form $f(x, c) = cx^{c-1} exp(-x^{c})$, for $x \ge 0$, $c > 0$. Data associated with (**c**) is best described by a Weibull max distribution that shows a probability density function of the form $f(x, c) = c(-x)^{c-1} exp(-(-x)^{c})$, for $x < 0$ and $c > 0$.

Figure 4a shows the best combination of parameters to control the virus transmission in local indoor places. The daily testing and 90% of random sample controls the transmission in 2 to 9 days. Its Spearman coefficient suggests a high and positive correlation between these parameters. Figure 4b shows the second best strategy, twice a week testing and 75% of random sample. It controls the spread of disease in 2 to 17 days. Its Spearman coefficient is lower than, and the scatter points show higher dispersion than, the first strategy. Figure 4c shows the third strategy, daily testing and 75% of random sample. This combination of parameters controls worse the transmission based on a large period of time. Its Spearman coefficient is lower than, and the scatter points show higher dispersion than, the first and second strategies. Finally, Fig. 4d shows the statistical distributions and best fits based on the number of testing samples of each of the above strategies. The least expensive strategy is the daily testing and 75% of random sample because of the number of cells tested. It shows a median of 181.68 tested cells or in monetary terms between $218,016.0–$1,362,600.0 mxn ($9629.04–$60,317.76 usd) (Table 1). The next least expensive is the daily testing and 90% of random sample. It shows a median of 194.47 tested cells or in monetary terms between $233,364.0–$1,458,525.0 mxn ($10,306.9–$64,564.0 usd). The most expensive strategy is twice a week and 90% of random sample. It shows a median of 284.24 tested cells or in monetary terms between $341,088.0–$2,131,800.0 mxn ($15,064.7–$94,367.6 usd).

**Table 1:** Statistics of testing-isolated strategies in the array of (10, 10).

n	Test interval	% rs	Best fit	Parameters	KS test	First moments
10,000	Daily	90	Beta	(9.0898, 2,598,074.4982, 17.7949, 53,890,486.4759)	(0.0050, 0.9629)	(199.4736, 206.3408, 3910.8787, 0.6633, 0.6600)
10,000	Daily	75	Weibull Min	(106.2499, 2191.0265, 325.4431, 13,174.2205)	(0.0063, 0.8213)	(284.2427, 283.0571, 3716.8704, − 0.0636, − 0.2631)
10,000	Twice a week	90	Weibull Max	(0.2251, 182.00, 1.6213)	(0.0042, 0.9930)	(181.6816, 104.7816, 726,484.8260, − 99.6032, 42,214.9272)

Percentage of random sample (% rs). Estimated parameters (a, b, loc, scale). KS goodness-of-fit test (D, p-value). First moments (median, mean, variance, skewness, kurtosis).

After analyzing these findings, we concluded that the first strategy is the best one. It controls the virus transmission in at least 1 week and the cost of the testing is slightly higher than the least expensive strategy. Then, in this scale of analysis, we identify the combination of parameters presented in Fig. 4a as the most desirable intervention in small workplaces.

**Figure 5:**
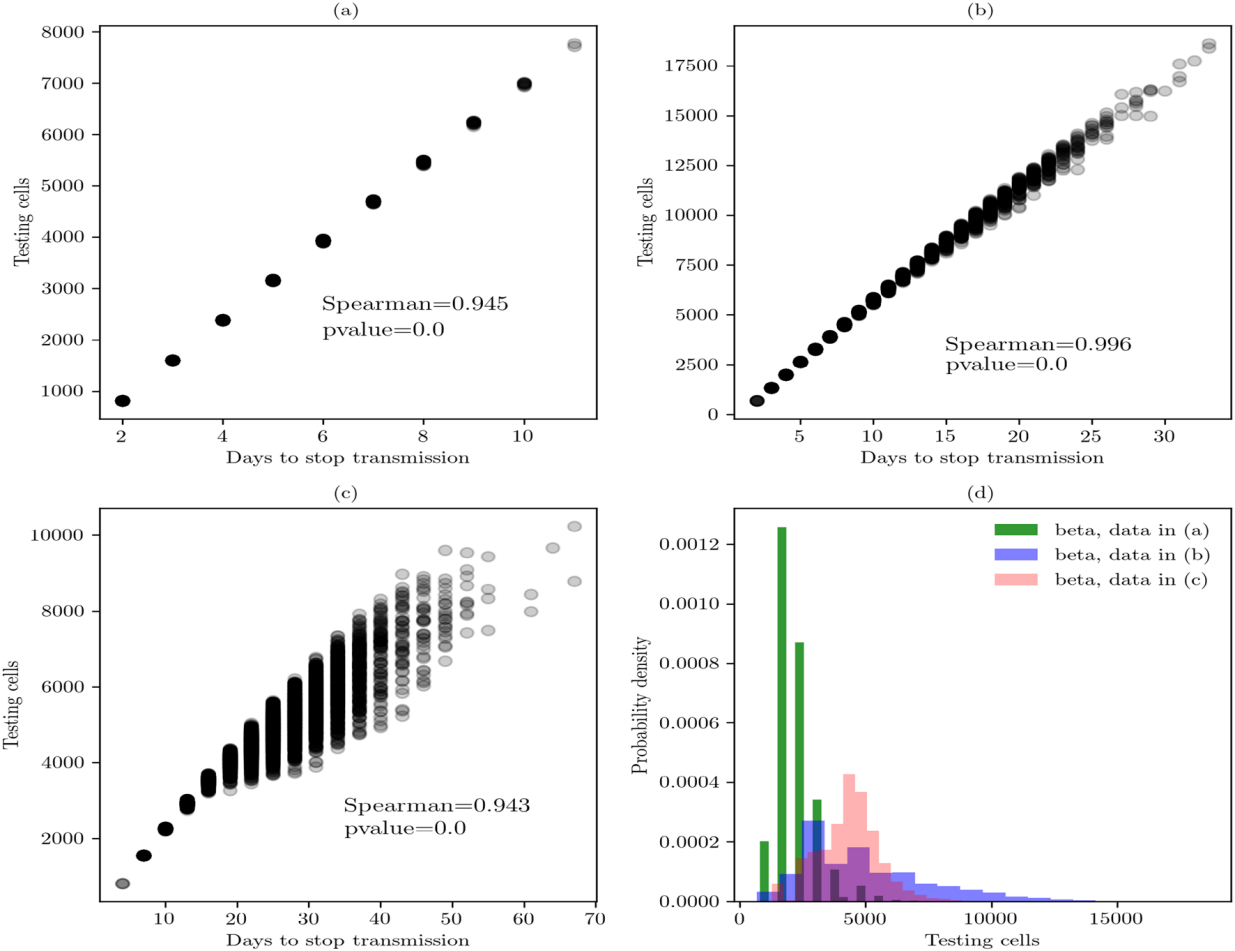
Sensitivity analysis based on scatterplots in the array of (30, 30). (**a**) Daily testing and 90% of random sample. (**b**) Twice a week testing and 90% of random sample. (**c**) Daily testing and 75% of random sample. (**d**) Distribution fitting of the cost for the testing strategy in the three cases. Data in (**a**), (**b**), and (**c**) are best described by a beta distribution that shows a probability density function of the form $f(x,a,b)=\frac{\gamma (a+b)x^{\alpha -1}(1-x)^{b-1}}{\gamma (a) \gamma (b)}$, for $0<x<1$, $a>0$, $b>0$.

Next, we present the findings related to the array size of (30, 30) (Fig. 5). Figure 5a shows the best combination of parameters to control the virus transmission in local indoor places. The daily testing and 90% of random sample controls the transmission in 2 to 11 days. Its Spearman coefficient suggests a high, positive correlation between these parameters. Figure 5b shows the second best strategy because it controls the spread of disease in 2 to more than 30 days—this is significantly higher than the past combination of parameters. Its Spearman coefficient is high and positive and suggests a strong correlation.

Figure 5c shows the third strategy in which the virus transmission was controlled on a larger period of time. Its Spearman coefficient is high and positive, and the scatter points show higher dispersion than the first and second strategies. Finally, Fig. 5d identifies the least expensive strategy of the three. This strategy is the daily testing and 90% of random sample. It shows a median of 2028.41 tested cells or in monetary terms between $2,434,092.0–$15,213,075.0 mxn ($107,505.7–$673,432.1 usd) (Table 2).

**Table 2:** Statistics of testing-isolated strategies in the array of (30, 30).

n	Test interval	% rs	Best fit	Parameters	KS test	First moments
10,000	Daily	90	Beta	(2.8583, 16.9468, 534.3316, 11,293.0408)	(0.0060, 0.8575)	(2028.4100, 2164.2004, 757,008.4565, 0.8468, 0.7654)
10,000	Daily	75	Beta	(2.9444, 90,294,701.0929, 605.5727, 134,405,606,190.0885)	(0.0051, 0.9536)	(4503.4733, 4988.4108, 6,523,948.4271, 1.1655, 2.0377)
10,000	Twice a week	90	Beta	(1531.3427, 60,095.0441, − 43,679.9023, 1,931,659.0267)	(0.0065, 0.7917)	(4309.6076, 4319.5363, 1,467,117.2611, 0.0491, 0.0035)

Percentage of random sample (% rs). Estimated parameters (a, b, loc, scale). KS goodness-of-fit test (D, p-value). First moments (median, mean, variance, skewness, kurtosis).

After analyzing these findings, we concluded that the first strategy is the best one. It controls the virus transmission in at least 1 week and the cost of the testing is the least expensive one. Therefore, that combination of parameters suggested the most desirable intervention in medium-sized workplaces.

**Figure 6:**
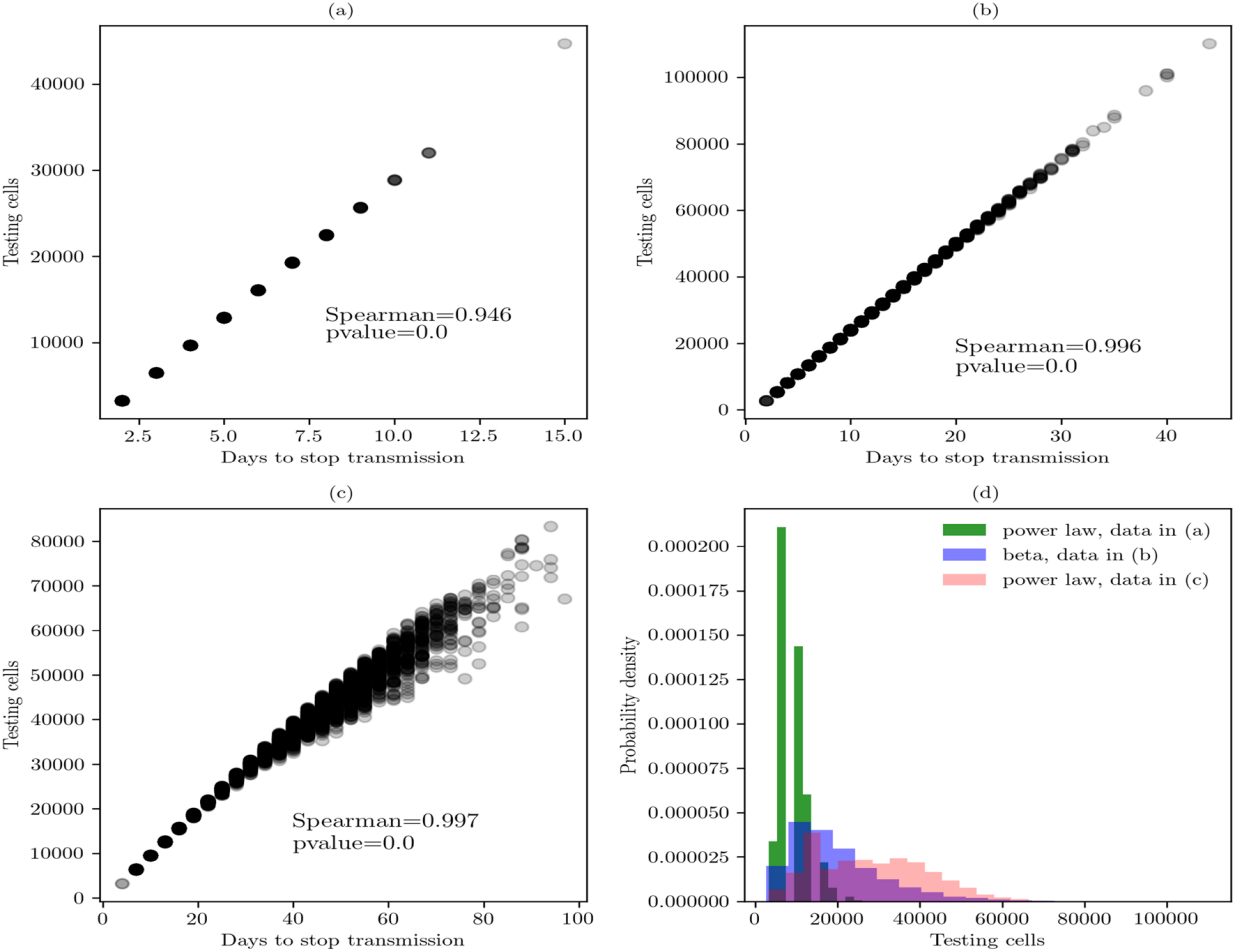
Sensitivity analysis based on scatterplots in the array of 60 × 60. (**a**) Daily testing-isolated and 90% of random sample. (**b**) Twice a week testing-isolated and 90% of random sample. (**c**) Daily testing-isolated and 75% of random sample. (**d**) Distribution fitting of the cost for the testing-isolated strategy in the three cases. Data in subfigures (**a**) and (**c**) is best described by a power law distribution that shows a probability density function of the form $f(x, \alpha ) = \alpha x^{\alpha -1}$, for $0 \le x \le 1$, $\alpha > 0$. Data in (**b**) is best described by a beta distribution that shows a probability density function of the form $f(x,a,b)=\frac{\gamma (a+b)x^{\alpha -1}(1-x)^{b-1}}{\gamma (a) \gamma (b)}$, for $0<x<1$, $a>0$, $b>0$.

Finally, we present the findings related to the array size of (60, 60) (Fig. 6). Figure 6a shows the best combination of parameters to control the virus transmission in local, but larger indoor places. The daily testing and 90% of random sample controls the transmission in approximately 15 days. Its Spearman coefficient suggests a high, positive correlation between these parameters. Figure 6b shows the second best strategy because it controls the spread of disease in more than 40 days. Its Spearman coefficient is high and positive suggesting a strong correlation. Figure 6c shows the third strategy in which the virus transmission was controlled in a larger period of time. Its Spearman coefficient is high and positive, and the scatter points show higher dispersion than the first and second strategies. Finally, Fig. 6d identifies the least expensive strategy of the three. This strategy is the daily testing and 90% of random sample. It shows a median of 14466.10 tested cells or in monetary terms between $17,359,320.0–$108,495,750.0 mxn ($766,703.3–$4,802,745.2 usd) (Table 3).

**Table 3:** Statistics of testing-isolated strategies in the array of (60, 60).

n	Test interval	% rs	Best fit	Parameters	KS test	First moments
10,000	Daily	90	Power law	(0.5275, 3239.9999, 41,768.5146)	(0.0067, 0.7491)	(14466.1018, 17665.0273, 1.56052589e+08, 0.5863, − 0.9234)
10,000	Daily	75	Beta	(2.9417, 16.7101, 1169.8513, 133,801.8123)	(0.0066, 0.7744)	(19600.5595, 21199.0635, 1.10342031e+08, 0.8243, 0.7094)
10,000	Twice a week	90	Power law	(0.6942, 3239.9999, 88,198.7279)	(0.0066, 0.7653)	(35737.5789, 39380.7156, 6.98309853e+08, 0.3260, − 1.1526)

Percentage of random sample (% rs). Estimated parameters (a, b, loc, scale). KS goodness-of-fit test (D, p-value). First moments (median, mean, variance, skewness, kurtosis).

These findings suggested that the first strategy is the best. It controls the virus transmission in around 2 weeks and the cost of the testing is the least expensive. Therefore, the combination of those parameters suggested the most desirable intervention in larger workplaces.

In summary, results showed that the best strategy in each of the array sizes is the combination of daily testing and 90% of a random sample. This strategy shows the least expensive testing intervention, but these costs are significantly higher than any cost that we have known. The impact of these findings is that the virus transmission can be controlled in local indoor workplaces. This local strategy can be replicated to urban areas covering the most vulnerable locations; for example communities of the most vulnerable individuals due to commorbidities, age, race, or income. Then, we can better organize the return to different activities based on a credible and intelligent testing approximation for saving lives and reactivating the economy.

## Discussion

Different interventions to reduce the SARS-CoV-2 transmission have been suggested by policy-makers around the world. Most of the time, these interventions have been ineffective. The main issue with a successful intervention is to generate a feasible and credible national strategy, based on science, where local communities and workplaces actively participate.

In this study, we proposed a local testing-isolating intervention for reducing the virus transmission in indoor workplaces to reopen and activate the economic system. Our formulation, based on the 2D CA and calibrated by official data of the Mexican epidemic, describes different scenarios that identify the most desirable testing time frequency and the most adequate random trails. However, these scenarios were not in line with our hypothesis. We expected that the time frequency of testing and the size of random trails depended on the size of the array, but they do not. Moreover, we believed that a value of 70% of people in random trails was enough to mitigate the virus transmission, but it is not. We were right about the economic cost of the testing strategies. Therefore, we found that, in all cases of array sizes, the best time frequency is daily and the best percentage of sampling is at least 90%. This combination of parameter values controls and stops the virus transmission in approximately a week. Compared with other scenarios, the economic cost is lower because the testing strategy is intensive in days and in size of random samples. The second best strategy is to apply the daily frequency and 75% of random samples. This strategy controls and stops the transmission in larger time steps and shows a larger number of infected cells before stoping the transmission. The economic cost is higher because the intensity of the testing strategy is in more time steps. Finally, the third strategy is related to the time frequency of twice a week and 90% of random samples. It is the most expensive strategy and the number of infected cells can be the total before controlling the transmission. If the goal of the strategy is to control and stop the spread of the virus, the testing time interval has not to be larger than twice a week.

We surprisingly identify that the economic cost of any intervention for controlling the SARS-CoV-2 transmission—or any other disease in the future—without a vaccine is higher than any other that we have known. Without a national plan that considers local communities and workplaces as the key to mitigate and decrease the virus transmission, we are doomed to repeat current mistakes. In particular, to establish the national health and the economic systems on political guidelines instead of science principles is a significant mistake that will accumulate overspending and lost of lives. Therefore, we suggest that our priorities have to be reorganized, where the health and the natural environment are above the economic interests.

Our results are encouraging and should be validated in real local communities and workplaces not only in Mexico, but also in other countries. Because of the lack of empirical analyses that apply approximately our intervention strategy, it is undetermined, up until now, to validate the operational process related to the model validation^64–67^. It is important to determine if our model outputs agree with observed data. Then, this represents a current gap between our findings and real scenarios. However, our conceptual validation based on the assumptions, simulation setting, and parameters can determine if our model is justifiable. Therefore, our formulation shows a high internal consistency between the theory, assumptions, initial conditions, and our expert intuition that produce reliable outputs.

We believe that an interdisciplinary approximation is appropriated to face the current health and economic costs. Therefore, we propose to apply the most intensive testing strategy to reopen the economy the safest way possible, minimizing the human cost, even if the economic cost is not the lowest. Therefore, the strategy we propose is the following:

1. The federal government has to invest in generating and distributing an efficient viral test. In particular, a test with less than 15% false positive and faster diagnose time, around 15 min.
2. To reopen activities, first responders and essential workers have to be grouped based on the physical installations they share for work. For example, healthcare workers are grouped based on the building they continuously use through the day. Then, each group of first responders has to be tested daily and at least 90% of its population.
3. Regardless if all tested negative or not for the virus, given that the probability to get infected and spread the virus does not depend on whether a person tested negative a week before, and given that the symptoms may appear within 14 days, we suggest to wait 1 week to start the testing-isolating strategy again for at least 90% of the population chosen randomly at a daily frequency.
4. The testing strategy needs to continue until a vaccine can be distributed and applied for the population. Moreover, this strategy can continue in the presence of reinfections or new variants and subvariants.

Finally, who is going to pay for the testing strategies? Each of us has to pay for it. The federal government has the constitutional obligation to give free access to any citizen for the healthcare system. Then, a revenue stream of the public sector has to pay at least 3/4 of the total cost of the test, and let the society coordinate its implementation and monitoring. The responsibility of the government is to provide and distribute an efficient test, to remove any type of bureaucratic and political barriers to distribute such a test, and to let the science guide us through the epidemic. However, by looking at official records on deaths and the need to convert hospitals designated to fight other type of diseases and illness for COVID-19, it is evident that the current inaction of the government exemplifies the failure of different prevention policies through time. It is not enough that the government focuses on mitigating the epidemic; science has to guide the path towards actually fighting the virus and stopping the spread. On the other hand, as society, we have the responsibility to face and answer unresolved mistakes. If we start late to make actions for future pandemics, we will accumulate costs sooner rather than latter. Therefore, we have to rethink and generate our prevention strategies in which science will lead us.

The circumstances of the current pandemic situation have showed that the COVID-19 testing is considerably decreasing in different countries, in particular in Mexico. The presence of this particular situation implies that it is very likely that our intervention strategy will not be considered in the short run. This is tragic and unacceptable because if someone, in particular kids, tested positive to COVID-19, the neurological complications are highly possible. As Chou et al.^68^, observe: “...neurological manifestations were found in approximately 80% of patients hospitalized with COVID-19.” Therefore, we strongly suggest further studies with more focus on implementing similar intervention strategy to decrease the number of infections not only by COVID-19, but also by other virus with similar mode of transmission such as the Ebola, smallpox, and tuberculosis.

## Conclusion

Our theoretical model produces reliable outputs due to the high internal consistency of our approach and the sensitivity analysis. It is fundamental to promote any type of intervention for controlling the SARS-CoV-2 transmission without a vaccine. Small-scale interventions are the best option because local communities can self-organize the testing and monitor infected cases. Local communities are the key to propagate the testing-isolating strategy at different scales. Therefore, first responders and essential workers are the basic elements to initiate waves of testing-isolating interventions that control the virus until a vaccine is deployed to everyone.

To honor and remember those people who lost their lives due to the COVID-19, we have to work closely with the scientific community for providing better-informed strategies to decision-makers and to coordinate global efforts for developing a solution for this unprecedented health and economic crisis.

## Data Availability

The source code and the material and findings data of this study are openly available in the OSF, project Testing SARS-CoV-2 strategies for reopening the economy (10.17605/OSF.IO/7E98T).
